# Subacute Disseminated Intravascular Coagulation in a Patient with Liver Metastases of a Renal Cell Carcinoma

**DOI:** 10.1155/2017/1023538

**Published:** 2017-04-05

**Authors:** L. C. van der Wekken, R. J. L. F. Loffeld

**Affiliations:** Department of Internal Medicine, Zaans Medisch Centrum, Zaandam, Netherlands

## Abstract

Disseminated intravascular coagulation (DIC) is a syndrome characterised by simultaneous bleeding and thromboembolic formation. Its acute form is associated with severe bacterial infections and hematological malignancies. It has a fulminant presentation with prolonged bleeding times and diffuse thrombosis. On the other hand, chronic DIC can be asymptomatic for long periods of time and can be seen in patients with disseminated malignancies. This case report describes a patient who developed DIC within one week and bled profusely from venipuncture wounds. An underlying hepatogenic metastasised renal cell carcinoma appeared to be the cause. This is an uncommon and diagnostically challenging presentation.

## 1. Introduction

Renal cell carcinoma (RCC) makes up for 2% of all new discovered malignancies and is the most common tumour of the kidney. Its incidence rate differs worldwide, with an incidence in Europe of 115 per 1000 European inhabitants [1]. In the year 2000, the United States had 31.200 new cases causing approximately 11.900 deaths. Males are at higher risk for RCC, especially in their 5th to 8th decade, with a median age of diagnosis of 66 years. The incidence is rising, probably due to improved imaging techniques, because mainly early stages are more often diagnosed. In 1970, 10% of the RCCs were diagnosed incidentally, compared to 61% in 1998 [2]. Its mortality rate depends on its stage, with a median 5-year survival rate of 62 percent. RCC predominantly metastasises to the lungs, retroperitoneal space, bones, and brain and sometimes to the liver. Treatment options consist of cryo- or radio frequent ablation, surgery, immunotherapy, or angiogenesis blockers [2].

In the present case, a patient with a very rare and strange complication of a renal cell carcinoma is described.

## 2. Case Report

A 70-year-old man was admitted to the Department of Internal Medicine and Gastroenterology, because of abnormalities seen on an abdominal ultrasound. The ultrasound was performed for follow-up, because of a renal cell carcinoma two years earlier, for which the patient underwent a nephrectomy. Histological investigation showed a pT2a tumour with angioinvasion. In the past, he had undergone a laparoscopic cholecystectomy because of symptomatic gallstones. He also had atrial fibrillation and a DDDR-pacemaker because of a high grade AV-block.

The ultrasound showed multiple echogenic areas in the liver, probably due to metastases. The patient complained of nausea, vomiting, and diminished toleration for larger portions of food. He was tired and experienced a weight loss of five kilograms in two weeks. He noted a few episodes of nose bleeding and had a tiny wound on his ankle that continued bleeding.

On physical examination, slight icteric sclerae, signs indicative of bilateral pleural effusion and ascites, were found. Laboratory findings are summarised in Table 1. There were no signs of active inflammation, given the fact that erythrocyte sedimentation rate and C-reactive protein were low. Blood cultures remained negative. The calculated DIC score by the International Society of Thrombosis and Haemostasis [3] was 6, which makes DIC probable.

**Figure 1:**
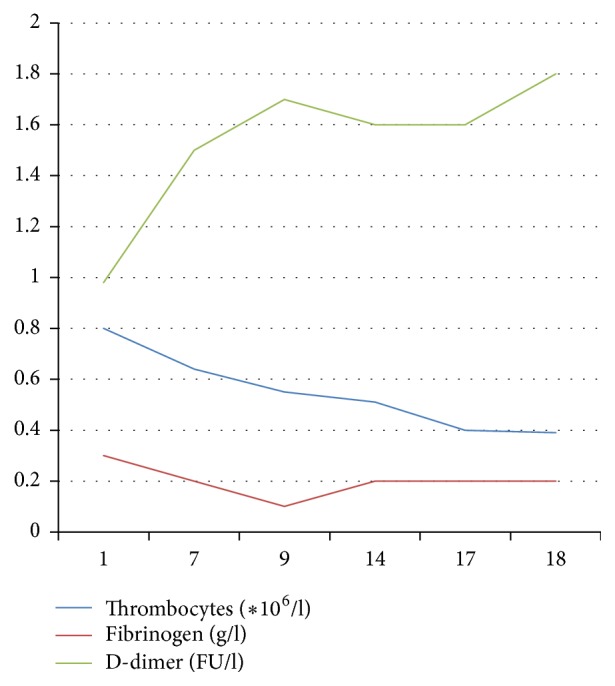
The course of coagulation abnormalities.

There were no signs of liver failure. Albumin and ATIII were within normal ranges. Patient normally used a coumarin derivate because of his atrial fibrillation. His INR was within the therapeutic range one week before admission, while using normal dosages of acenocoumarol. He had stopped the anticoagulation in preparation for a liver biopsy. The course of the coagulation abnormalities is shown in Figure 1 [4].

CT scan of thorax and abdomen confirmed the findings of the ultrasound. A FDG-PET was performed, but no high uptake as sign of infection or tumours could be identified. Due to the existing diffuse intravascular coagulation with a high risk of bleeding, the diagnostic liver biopsy could not be performed.

The clinical situation rapidly worsened and the patient died four weeks after admission.

Postmortem examination showed multiple petechia and purpura on the thoracal skin, signs of a recent myocardial infarction and an enlarged liver, weighing 5100 g, no splenomegaly, and no signs of infection. The majority of the liver consisted of tumorous areas, without distinct borders. The largest tumour was measured at 19 cm. A tubulopapillary growing pattern was identified within the tumour. The tumour cells had hyperchromatic nuclei and a large quantity of eosinophilic cytoplasm. Immunohistochemical tests revealed positivity of the tumour cells for pankeratin, CK19, vimentin, CD10, and PAX8. The tumour cells were negative for CK7 and CK20. Histologically, this pattern is suggestive for renal cell carcinoma. When compared to the renal cell carcinoma which has been excised earlier in the patient's life, the tumorous cells in his liver showed a great resemblance.

It could be concluded that this patient had diffuse intravascular coagulation with severe hypofibrinogenemia of very short duration, due to a hepatogenic metastasised renal cell carcinoma.

## 3. Discussion

### 3.1. Clinical Characteristics

Disseminated intravascular coagulation (DIC) is a syndrome characterised by bleeding, thrombosis, or both. Laboratory investigation of patients with DIC showed signs of activation of both the clotting and the fibrinolytic system [3, 4].

The clinical presentation depends on the underlying disease triggering DIC. Acute DIC is associated with (bacterial) infections and sepsis, crush injuries, hematologic malignancies (especially promyelocytic leukemia), and obstetric complications. Patients with acute DIC may present with petechiae, purpura, and bleeding from wounds and venipuncture sites. Although less obvious, microvascular thrombosis (and sometimes large vessel thrombosis as well) may also occur. Organ systems most often affected are the skin, lungs, kidneys, pituitary, liver, and adrenals [3, 5, 6].

In contrast, chronic DIC, most often seen in patients with an underlying malignancy, has a less fulminant presentation. It is characterised by a more gradual, chronic, and systemic activation of the coagulation system. Exhaustion of platelets and coagulation factors may lead to bleeding. Thrombocytopenia and elevated levels of fibrin-related markers can be found. Chronic DIC commonly presents with gingival bleeding, easy and spontaneous bruising, and bleeding from gastrointestinal and urogenital tract. Also diffuse or singular thrombosis is seen. Malignancies of the gastrointestinal tract, pancreas, prostate, and lung are associated with chronic DIC [6]. In a cohort study conducted by Levi, 5,2% of the patients with chronic DIC turned out to have a renal cell carcinoma [7].

In a cohort of 1117 patients with solid tumours, 76 (6,8%) turned out to have chronic DIC, and almost half of them (46%) had disseminated cancer to the liver [7]. Another study showed that approximately 75% of patients with disseminated malignancies have laboratory evidence for DIC, of which 25% will eventually become clinically manifested [6]. Patients with cancer-related DIC have a significantly lower survival when compared to patients with malignancies but without DIC [7].

### 3.2. Pathogenesis

Tissue factor plays a crucial part in clotting. It binds factor VII(a) to activate factors IX and X and therefore promote coagulation [5]. Solid tumours are found to express tissue factor, which is needed in angioneogenesis and metastasising [8]. Also chemotherapeutics and angiogenesis blockers are associated with an increased risk of venous and arterial thrombosis, probably due to the harmful effect on the endothelial lining of the vasculature [9]. Activated endothelial cells also produce PAI-1, which is an inhibitor of plasminogen activation. Therefore, endothelial dysfunction leads to diminished activation of fibrinolysis therefore contributing to the favouring of coagulation [5]. Endothelial cells in cancer patients get activated by cytokines after which they show enhanced TF expression and TF-dependent procoagulant activity [10]. IL-6 is a cytokine associated with this response, whereas IL-10 has the ability to inhibit DIC [5].

DIC in cancer patients is also promoted by a protein called cancer procoagulant, which is a cysteine protease with factor X activating properties and is found in patients with solid tumours [11]. Some mucinous adenocarcinomas have the ability to activate factor X to factor Xa in a nonenzymatic way to contribute to coagulation [4].

Besides coagulation, fibrinolysis also plays an important part in DIC. Endothelial cells produce, storage, and release tissue-type plasminogen activator and urokinase-type plasminogen activator, which are fibrinolytic activators.

### 3.3. Treatment

The treatment of DIC is to treat the underlying disease. In patients with cancer-related DIC, the disorder resolves when the cancer is brought into remission [5]. An experimental study showed a beneficial effect of unfractioned heparin in lipopolysaccharide-induced hypercoagulability [12], but it is not generally used to prevent thrombus formation in patients with DIC.

Although the benefit of low molecular weight heparin (LMWH) usage in patients with DIC is uncertain, acutely ill patients, patients with cancer, and older patients are at risk for venous thromboembolism, so when admitted to the hospital, prophylactic treatment with LMWH is indicated [13].

In patients with a platelet count <50 × 10^9^/l and bleeding complications or who have to undergo an invasive procedure, platelet transfusions are indicated. When no such urgent circumstances are present, a platelet count <10–20 × 10^9^/l is generally used as threshold for platelet transfusions. Due to the fact that DIC in cancer is partly caused by a lack of fibrinolysis, one should not use antifibrinolytic agents (e.g., tranexamic acid) [5, 14].

## 4. Conclusion

In this case, a 70-year-old man with bleeding diatheses due to a hyperfibrinolytic state caused by DIC related to a renal cell carcinoma was presented. Cancer-related DIC is characterised by a chronic and gradual activation of the coagulation system, with thrombosis and mild bleeding as the key symptoms. In this patient, a more rapid decline in clotting factors and profuse bleeding were seen, which is an uncommon presentation, which leads to diagnostic and therapeutic dilemmas.

## Figures and Tables

**Table 1:** Laboratory findings and blood drawn at admission.

	Value	Reference
Hemoglobin	8,0 mmol/l	8,5–11 mmol/l
Thrombocytes	80*∗*10^9^/l^*∗*^	150–400*∗*10^9^/l
Leukocytes	10,8*∗*10^9^/l	4–10*∗*10^9^/l
Schizocytes	Present	Not present
ALAT	138 IU/l	<40 IU/l
Bilirubin	35 *μ*l/l	<21 *μ*l/l
GGT	1421 U/l	<55 U/l
AF	438 U/l	<120 U/l
LDH	421 U/l	<250 U/l
PTT	Unmeasurable	
APTT	Unmeasurable	
Fibrinogen	0,3 g/l	2,0–4,0 g/l
D-dimer	0,98 FU/l	<0,5 FU/l
Albumin	39 g/l	35–52 g/l
ATIII	112%	>70%
ESR	2 mm after 1 hour	<15 mm after 1 hour
CRP	11 mg/l	<5 mg/l

^*∗*^Non-EDTA sensitive.
